# PanWeb: A web interface for pan-genomic analysis

**DOI:** 10.1371/journal.pone.0178154

**Published:** 2017-05-24

**Authors:** Yan Pantoja, Kenny Pinheiro, Allan Veras, Fabrício Araújo, Ailton Lopes de Sousa, Luis Carlos Guimarães, Artur Silva, Rommel T. J. Ramos

**Affiliations:** Institute of Biological Sciences, Federal University Pará, Belém, Pará, Brazil; Universite Paris-Sud, FRANCE

## Abstract

With increased production of genomic data since the advent of next-generation sequencing (NGS), there has been a need to develop new bioinformatics tools and areas, such as comparative genomics. In comparative genomics, the genetic material of an organism is directly compared to that of another organism to better understand biological species. Moreover, the exponentially growing number of deposited prokaryote genomes has enabled the investigation of several genomic characteristics that are intrinsic to certain species. Thus, a new approach to comparative genomics, termed pan-genomics, was developed. In pan-genomics, various organisms of the same species or genus are compared. Currently, there are many tools that can perform pan-genomic analyses, such as PGAP (Pan-Genome Analysis Pipeline), Panseq (Pan-Genome Sequence Analysis Program) and PGAT (Prokaryotic Genome Analysis Tool). Among these software tools, PGAP was developed in the Perl scripting language and its reliance on UNIX platform terminals and its requirement for an extensive parameterized command line can become a problem for users without previous computational knowledge. Thus, the aim of this study was to develop a web application, known as PanWeb, that serves as a graphical interface for PGAP. In addition, using the output files of the PGAP pipeline, the application generates graphics using custom-developed scripts in the R programming language. PanWeb is freely available at http://www.computationalbiology.ufpa.br/panweb.

## Introduction

Next-generation sequencing (NGS) platforms have made major advances in DNA sequencing methods, mainly due to their increased yield and accuracy and their significantly reduced cost [1,2]. Due to NGS technologies, there has been an exponential increase in the number of complete genomes deposited into public databases such as the Online Genome Database (https://gold.jgi.doe.gov/). With the large number of available genomes, especially prokaryotic genomes, it has become possible to perform comparative analyses, such as pan-genomic analyses [3,4].

Comparisons of the genetic repertoires of organisms can aid in the discovery of genes of biotechnological, biomedical and environmental interest [5]. This approach can chart the occurrence of evolutionary events and can establish phylogenetic relationships [6]. Using comparative genomics, it is possible to investigate several genomic characteristics that are intrinsic to certain species [7]; using a pan-genomic approach, in which several organisms of the same species or genus are compared to identify similarities between the genomes, one can elucidate the virulence mechanisms of pathogenic organisms [8].

In addition, comparative genomics can be used with microorganisms that have different lifestyles to correlate their gene repertoires and genome sizes, as intracellular pathogens often undergo reductive evolution and gene loss [9].

In eukaryotes, comparative genomics has been used to identify homologs of human genes that have been linked to different types of diseases (cardiovascular, visual, auditory, endocrine and bone diseases) in model organisms such as *Drosophila melanogaster*, thereby making it possible to test new gene therapies [10, 5].

Among the software tools that are available to perform pan-genomics analyses, Panseq (Pan-Genome Sequence Analysis Program) [11] determines the core and accessory regions from a collection of genomic sequences based on user-defined parameters and also detects SNPs (single-nucleotide polymorphisms) in the core regions. However, Panseq is not capable of performing pan-genomic profile analyses or functional enrichment analysis [11, 12]. PGAT (Prokaryotic Genome Analysis Tool) [13] is a web database designed to compare genetic content among many microbial genomes; however, PGAT currently provides limited results for species in its database, and it does not support user-provided genomic data analysis [13, 12]. Finally, PGAP (Pan-Genome Analysis Pipeline) [12] integrates several functional models and can be used to study the evolutionary history of bacteria to find pathogenic mechanisms and to prevent and control epidemics; however, PGAP was developed using the Perl script language, and the pipeline needs to be installed, configured and parameterized, making it difficult to be used by users without computational experience. In addition, its output files are complex and difficult to interpret [14].

In this study we describe the development of a web platform to provide a graphical interface for PGAP that simplifies the analysis and incorporates new graphics that can aid in the interpretation of results.

## Materials and methods

### Implementation

PanWeb was implemented using the programming languages PHP (http://php.net/)) and R (https://www.r-project.org/), which were used to process the output results of PGAP and to generate graphs to facilitate data interpretation. In addition, we used other technologies, including hypertext markup language (HTML) version 5 to mark the site structure, cascading style sheets (CSS) version 3 to define the style and appearance of the site, and the JavaScript programming language.

### Pan-genomic analysis

The PGAP tool was chosen for use at the back end of the PanWeb platform, as PGAP presents installation and configuration challenges in that the pipeline uses several extra software tools in its analyses and can be difficult to use. For example, PGAP relies on UNIX terminals and employs an extensive command line that contains many parameters to be executed. In the pan-genomic analyses conducted in PGAP, orthologous and paralogous genes can be identified in addition to the accessory genome, central genome and species-specific region definitions. The cluster analysis of functional genes is one of the essential steps in the search for orthologs and paralogs among multiple genomes. For this purpose, PGAP uses two methods, namely MP (MultiParanoid) and GF (gene family), to conduct the analysis [12].

The MP method uses two algorithms, namely InParanoid and MultiParanoid [15]. First, the InParanoid algorithm performs a search for orthologs between each pair of strains, and the paralogs in each strain are considered by using BLAST to search for homologs in their own genomes. Next, the MultiParanoid algorithm performs the search for gene clusters among multiple strains [12].

The GF method uses BLASTALL among the mixed protein sequences, and the clustering process is performed by the MCL algorithm [16,17].

The minimum score value and *e-value* in BLAST are 40 and 10–8 for both methods (GF and MP) [12]. In order to reduce the time spent to Blast search, the PGAP script was changed to run the Diamond software [18], which is faster than Blast, and represented one of the main slower steps of PGAP pipeline.

### Pipeline

PanWeb receives as input the annotation files for each genome in EMBL format, and the web application then automatically generates the NUC and PEP files, which contain the coding DNA sequence (CDS) in nucleotide format and protein format, respectively; it also generates the FUNC file, which contains the product of each CDS. These files are subsequently processed by PGAP [12]. To avoid bias in the analyses, it is important to standardize the prediction and annotation of genomes by using the same annotation tool for all genomes. After running PGAP, PanWeb will process the results and will present the graphs that were obtained from the output files generated by the pipeline.

### Graphs for analyses

After PGAP processing, graph generation was implemented using R (https://www.r-project.org/). Table 1 describes each of the graphs.

**Table 1:** Name and description of each graph.

Graph	Description
Pan/core genome	Presents the pan-genome and core-genome profiles.
Vertical bar graph (blue)	Graph with the number of orthologous and paralogous genes shared among *n* strain combinations.
Horizontal bar graph	Graph showing the number of unique genes in each strain, i.e., strain-specific genes.
Pie charts	Displays the proportion of homologous genes shared among *n* strains.
Phylogenetic trees graphs	Graphs are generated from 3 algorithms: neighbor-joining, UPGMA and maximum likelihood (ML).

#### Pan-genome and core-genome graphics

The pan-genome (boxplots in blue) and core-genome (boxplots in red) graphs are generated from the file *2*.*PanGenome*.*Data*.*txt*. The boxplots represent the many possible combinations among all samples (all combinations of all sequenced genomes). In each combination, the genome of an organism is compared to the genome of another organism, and using score alignments, it is possible to infer which genes are orthologous and paralogous among the different strains. The combinations start on 1 (first graph boxplot) and continue up to the number of samples (combinations of 1 in 1, 2 in 2, 3 in 3 and so on). As the number of samples increases in the different combinations, a function (fitting functions to data points) can be fitted to the data set (boxplots) in a process known as regression. The graph also shows two curves (green and yellow) that are adjusted for the pan-genome and the core-genome. These curves are adjusted according to Heap’s Law, as represented by the formula [4]:

$$n=kxN^{-\alpha }$$ (1)

where *n* is the expected number of genes in the pan-genome or core-genome for a given number of genomes (*N*). The coefficients *k* and *α* (alpha) are calculated according to the fitting curve using the statistical computing language R. The curve fit can be performed for both the median (green curve) and the mean (yellow curve) of each distribution. In 2008, Tettelin and colleagues used Heap’s Law to infer whether a particular pan-genome would be open or closed. Thus, if α (alpha) < = 1, it can be inferred that the genome is question is an open pan-genome. In contrast, α (alpha) > 1 represent a closed pan-genome.

#### Bar graphs

The vertical bar graph (blue) displays the number of orthologs and paralogs that are shared among strains. The number *n* (1, 2, 3, 4,…, *n*) on the x-axis shows the clusters that are shared by *n* strains. Thus, the number 1 shows the number of unique genes found in each strain of the species, while the number *n* represents the core-genome. The graph was created using R software, employing the *1*.*Orthologs_Cluster*.*txt* file generated by PGAP.

A horizontal bar graph was generated with the aim of showing the number of unique genes in each strain. The *1*.*Gene_Distribution_By_Conservation*.*txt* file was used as the input to generate this graph with R software.

#### Pie charts

The pie chart shows the proportion of orthologs and paralogs that are shared among *n* (1, 2, 3, 4,…, *n*) strains and the contribution of each part with respect to the total, while also emphasizing the individual parts that correspond to the homologs that are shared among *n* strains.

#### Phylogenetic tree graph

PanWeb presents several phylogenetic trees that are created by PGAP based on two types of data. The first includes a gene distance matrix and the nucleotide sequences of the core gene clusters. The second includes the mutation and indel variations in the core gene clusters. Two algorithms, namely neighbor-joining (NJ) and UPGMA, are used for the construction of phylogenetic trees based on the two types of data. However, the maximum likelihood (ML) algorithm is used only for the mutation and indel variation data [12].

### Biological dataset

The *Escherichia coli* was used as a model. Its genomes are available at NCBI (http://www.ncbi.nlm.nih.gov/) under accession numbers AP014857, CP007025, FM180568, FN554766, CP009166, CP000247, CU928145, CP001671, NZ_CP007442, CP006830, NZ_LSZR000, CP004009, CP000946, CP005998, CP001509, CP001665, NZ_CP014268, CP014269, CP000819, CP009273, CP001396, CP001637, AP012030, CP000800, CP010344, CU928162, CP009644, FN649414, CP009578, LM993812, LM995446, CP000802, HF572917, CU928160, CU928164, CP001969, CP016038, LT601384, CP006784, CP014316, LN832404, CP011343, CP011342, CP014272, CP014270.

The parameters used to run PanWeb with the biological dataset were GF method, *e-value* of 0.00001, identity of 0.5 and coverage value of 0.5.

### Output files

Some output files are also of great importance in a pan-genomic analysis along with graphics, like *CDS*.*variation*.*for*.*evolution*.*txt*, that is generated in phylip software (http://evolution.gs.washington.edu/phylip.html) format and has information of the variations present in the core genome. The file contains the output of the 3 nucleotides corresponding to the amino acid where a variation was located. The file *Ortholgs_Cluster_Function*.*txt* contains the COG classification for each sample cluster, if a cluster have no COG classification, the file will be filled with the "-" character at the location of the COG identifier.

## Results and discussion

### PanWeb

The PanWeb graphical interface allows the submission of data to the comparative genomics pipeline. After each submission, a unique identifier is generated for the process, enabling the user to access the result through the search form on the homepage. PanWeb has shown to be efficient for a pan-genomic analysis and its user friendly interface presents the results in the form of graphs or tables, unlike the other tools available for pan-genomic analysis, such as Panseq [11] and PGAT [13], which is useful to users without computation skills.

The analysis page consists of two input types, an upload form to receive the input files in EMBL or GBK format and if the user wants to perform pan-genomic analysis with microorganisms that have COG classification, there is a list of available microorganisms In addition, there are other fields to define parameters, such as *e-value*, identity, coverage and type of analysis to be performed (Pan-genome profile analysis, Genetic variation analysis, Species evolution analysis or Function enrichment analysis). PanWeb also allows the user to choose between the MP and GF methods. There is an instructions section on the website that allows the user to review the resources available in the PanWeb interface.

### Analysis of *Escherichia coli* dataset

To evaluate the PanWeb website, 45 genomes of the genus *Escherichia coli* were used to perform an analysis which took 55 hours and 37 minutes.

**Fig 1:**
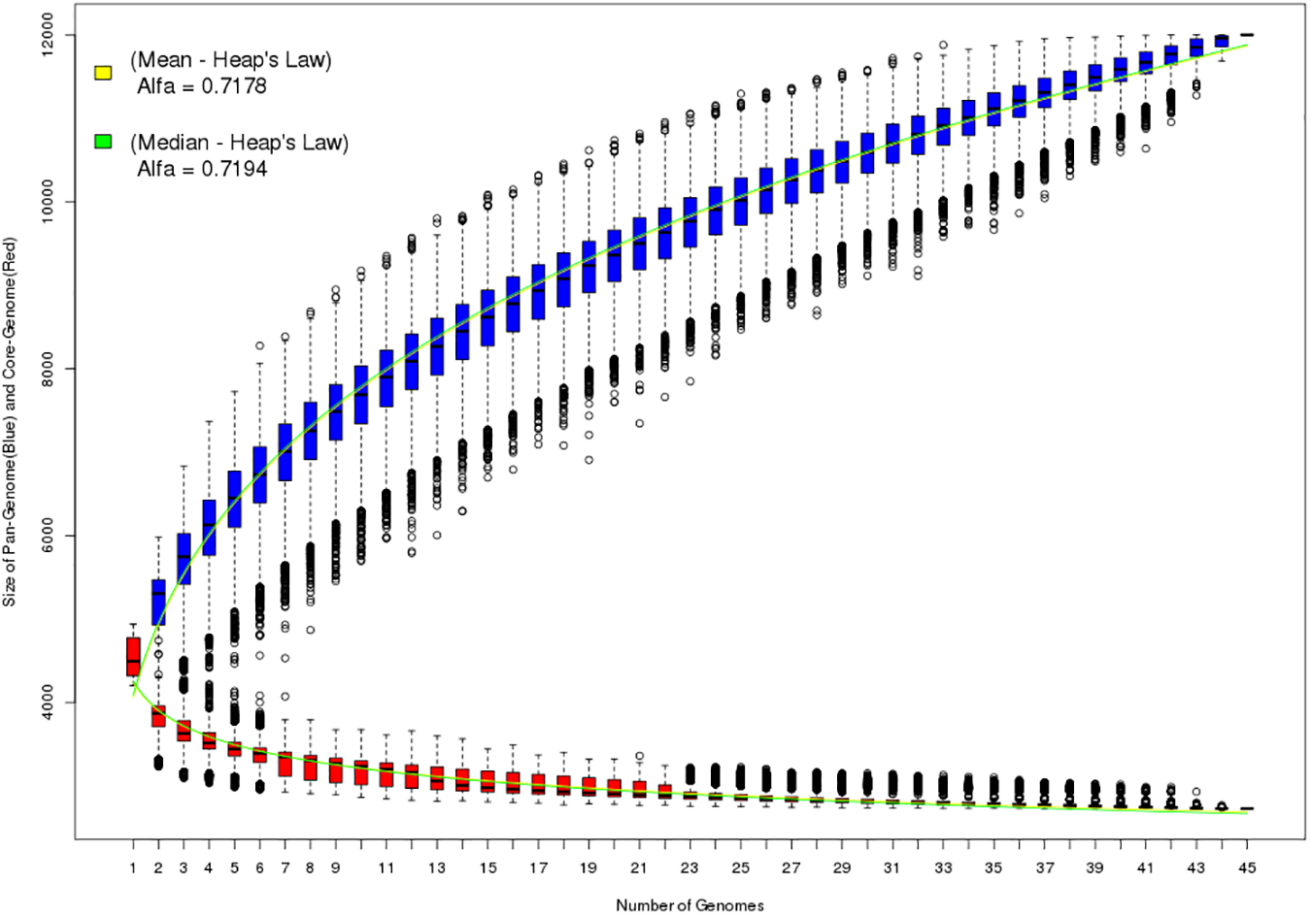
Pan-genome and core-genome. Graph representing the pan-genome (blue) and core-genome (red) of the 45 analyzed genomes. The graph also shows the α coefficient value of Heap’s Law when fitting the curve to the mean (yellow curve) or median (green curve) of the different boxplots.

The graph shown in (Fig 1) displays the pan-genome (blue boxplots) and the core-genome (red boxplots) of the genus *Escherichia coli*; it also shows the Heap’s Law curve fit using the medians (green curve) and means (yellow curve) of the distributions. The alpha exponent of Heap’s Law can be used to infer whether a pan-genome is open or closed; in this example, the alpha values were approximately equal to 0.71 (for both the curve fitting to the mean and the distribution fitting to the median), thus indicating an open pan-genome.

The graph in S1 Fig presents the number of paralogous and orthologous genes shared among *n* (45, 44, 43, 42,…, 1) strains. The vertical bar at number 45 on the x-axis represents the total number of genes shared among all 45 strains (i.e., the core-genome) in this analysis. The vertical bars from numbers 2 to 44 display the number of genes in the accessory or dispensable genome; the bar at number 2 shows the number of genes shared between two strains, the bar at number 3 shows the number of genes shared among three strains, and so on, until the bar at number 44. The vertical bar at number 1 represents the number of unique genes found among the 45 strains.

The graph shown in S2 Fig displays the number of unique genes for each evaluated strain, allowing the user to determine which strains have larger or smaller numbers of unique genes. In this evaluation, the strains *Escherichia coli albertii* KF1, *Escherichia coli* ED1 and *Escherichia coli* O127:H6 E2348/69 had the greatest number of unique genes relative to the other strains in the sample.

**Fig 2:**
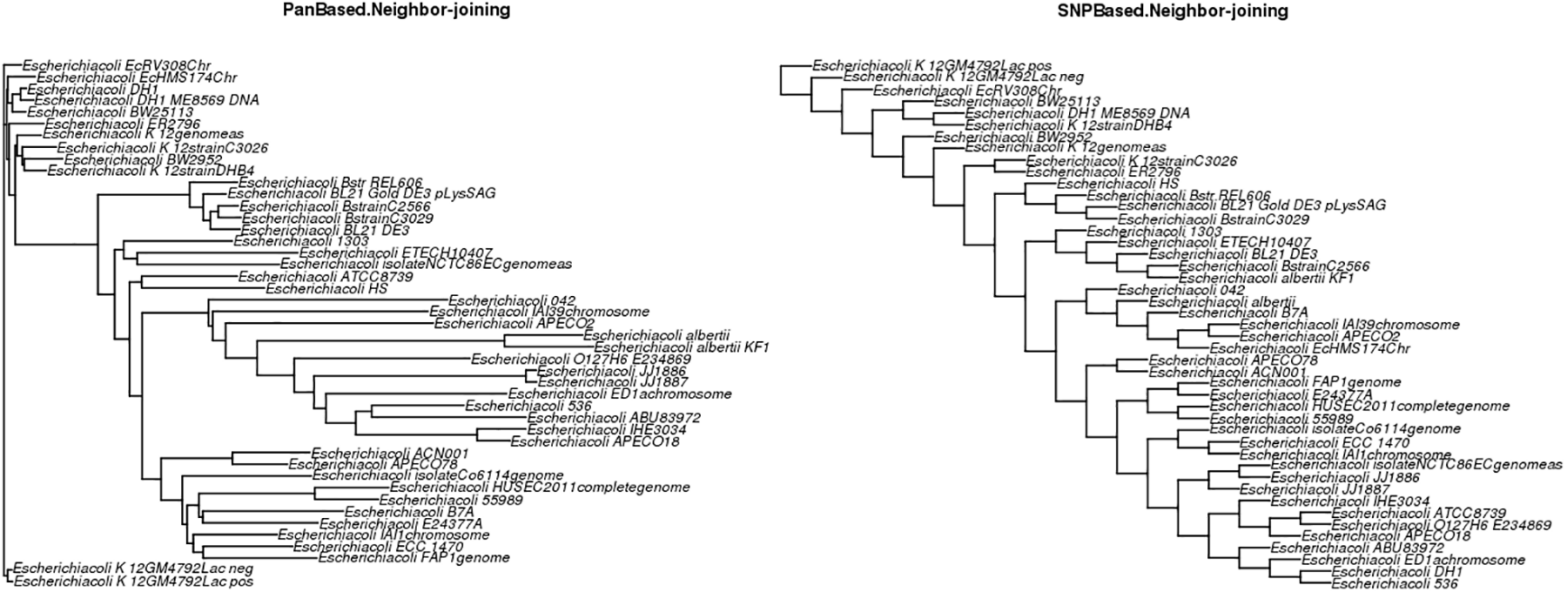
Phylogenetic trees based on the NJ algorithm. Phylogenetic trees showing species evolution analysis for the 45 strains based on the NJ algorithm. The left graph is based on the gene distance matrix for core gene clusters, and the right graph is based on indel variations in core-gene clusters.

The pie charts in S3 Fig show the proportions of orthologs and paralogs shared among the *n* strains. There were 2,726 genes shared among all strains (Core-genome) and 3,780 genes present only in single strains (i.e., the total number of unique genes observed in each strains in this analysis). For species evolution analysis, we evaluated five different types of graphs that were obtained through.tree files generated by PGAP. The graphs shown in (Fig 2) shows two phylogenetic trees, the first using the NJ algorithm to process the gene distance matrix and the nucleotide sequences in core gene clusters in the pan-genome (PanBased.Neighbor-joining) and the second to assess the mutation and indel variations in core gene clusters (SNP.Based.Neighbor-joining).

The graphs in S4 Fig present species evolution analyses that are also based on the two types of data (i.e., gene distance matrix and indel variations) but that use the UPGMA algorithm for constructing phylogenetic trees. Thus, the user can evaluate similarities and differences between the UPGMA and NJ algorithms for the same data set. For example, the strain *Escherichia coli* EcRV308Ch was located in an isolated cluster in the PanBased.Neighbor-joining graph (Fig 2); in the PanBased.UPGMA graph (S4 Fig), the same strain was located in a cluster with strains *Escherichia coli* K-12GM4792Lac- *and Escherichia coli* K-12GM4792Lac+.

The Maximum Likelihood (ML) algorithm is also used for species evolution analysis and uses only indel variation data (S5 Fig).

## Conclusions

The PanWeb platform provides a graphical interface that enables rapid analysis of the results obtained by PGAP software without the need for installation and configuration of software and packages used by the pipeline. In addition, it does not require an extensive command line to perform the pretreatment of input files (.pep,.nuc and.func files). As the results of PGAP are all presented as text files, PanWeb allowed the visualization of these results in a graphical format, thereby facilitating analysis.

## Supporting information

S1 Fig Vertical bar graph. Bar graph representing the number of orthologous and paralogous genes shared among the *n* strains that were analyzed.
(TIFF)

S2 Fig Horizontal bar graph. Bar graph showing the number of unique genes found in individual strains in the sample.
(TIFF)

S3 Fig Pie charts. Pie charts showing the proportion of homologous genes shared among the *n* strains.
(TIFF)

S4 Fig Phylogenetic trees based on the UPGMA algorithm. Phylogenetic trees showing species evolution analysis for the 45 strains based on the UPGMA algorithm. The left graph is based on the gene distance matrix for core gene clusters, and the right graph is based on indel variations in core-gene clusters.
(TIFF)

S5 Fig Phylogenetic trees based on ML algorithm. Phylogenetic tree showing species evolution analysis for the 45 strains based on the ML algorithm. The species evolution analysis is based on indel variations in core-gene clusters.
(TIFF)
