# Supplementary figures and images for: PanWeb: A web interface for pan-genomic analysis
Source: PLoS One. 2017 May 24;12(5):e0178154. doi: 10.1371/journal.pone.0178154 (PMC5443543; doi:10.1371/journal.pone.0178154)

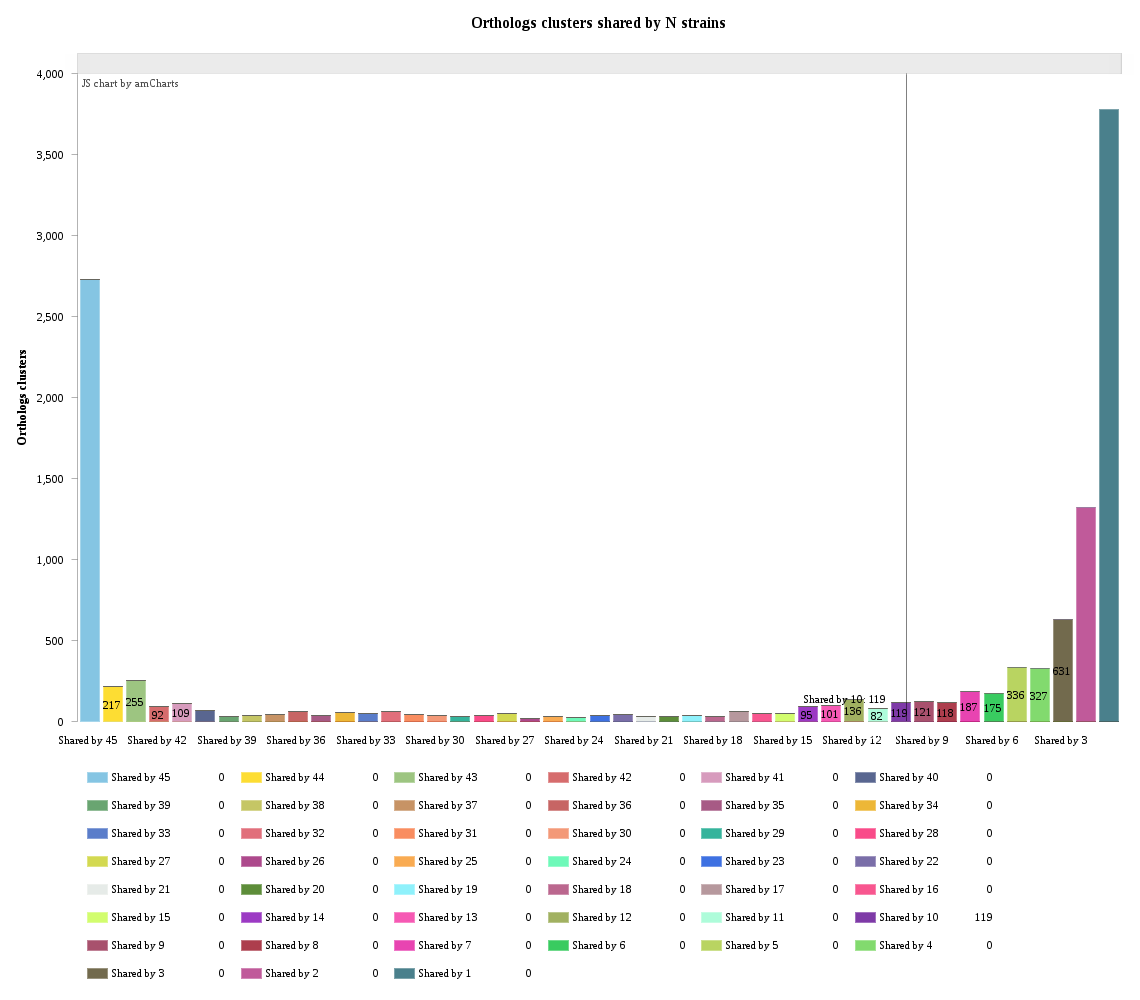

Supplement: S1 Fig — Bar graph representing the number of orthologous and paralogous genes shared among the n strains that were analyzed. (TIFF) [file pone.0178154.s001.tiff]

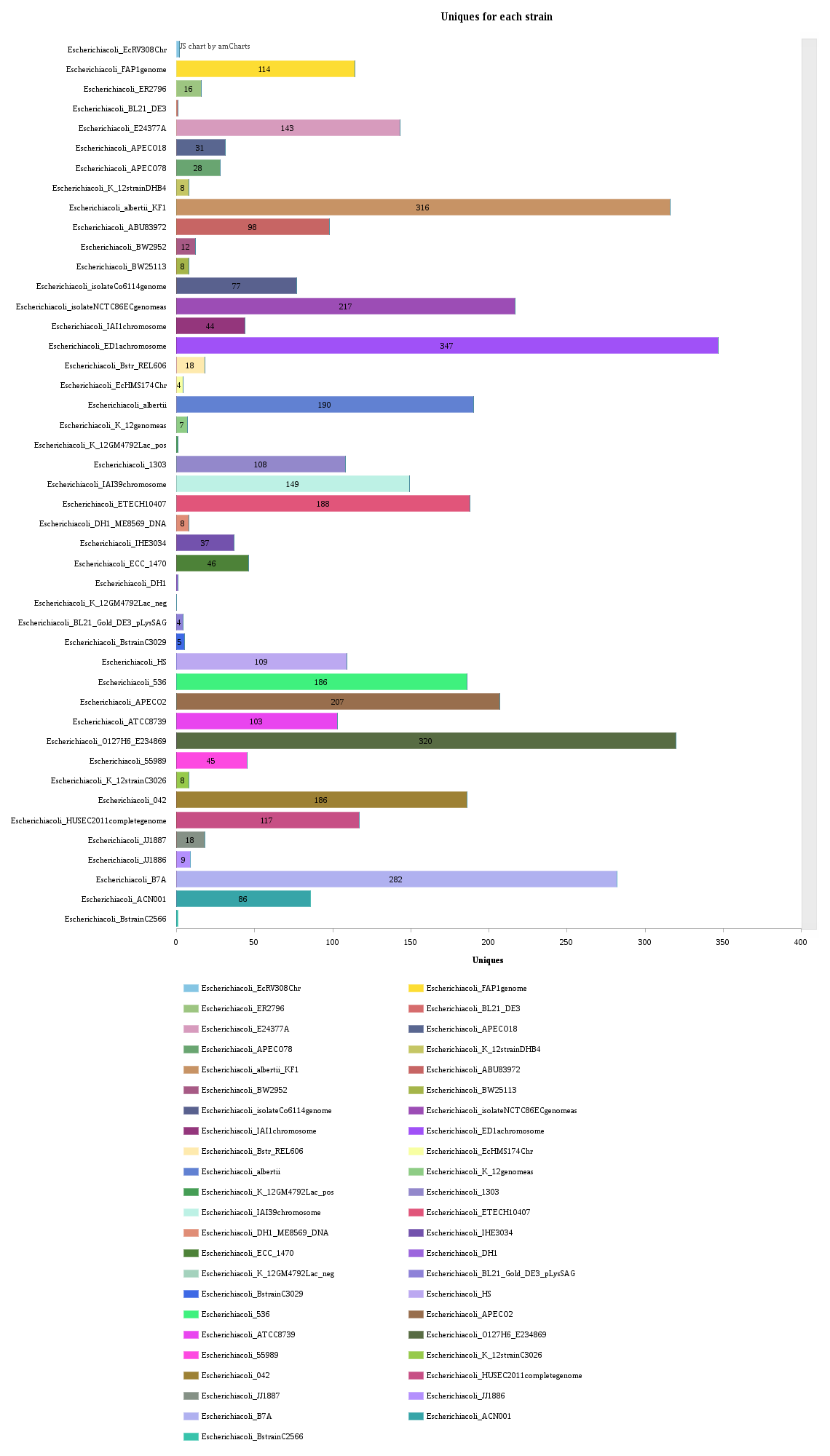

Supplement: S2 Fig — Bar graph showing the number of unique genes found in individual strains in the sample. (TIFF) [file pone.0178154.s002.tiff]

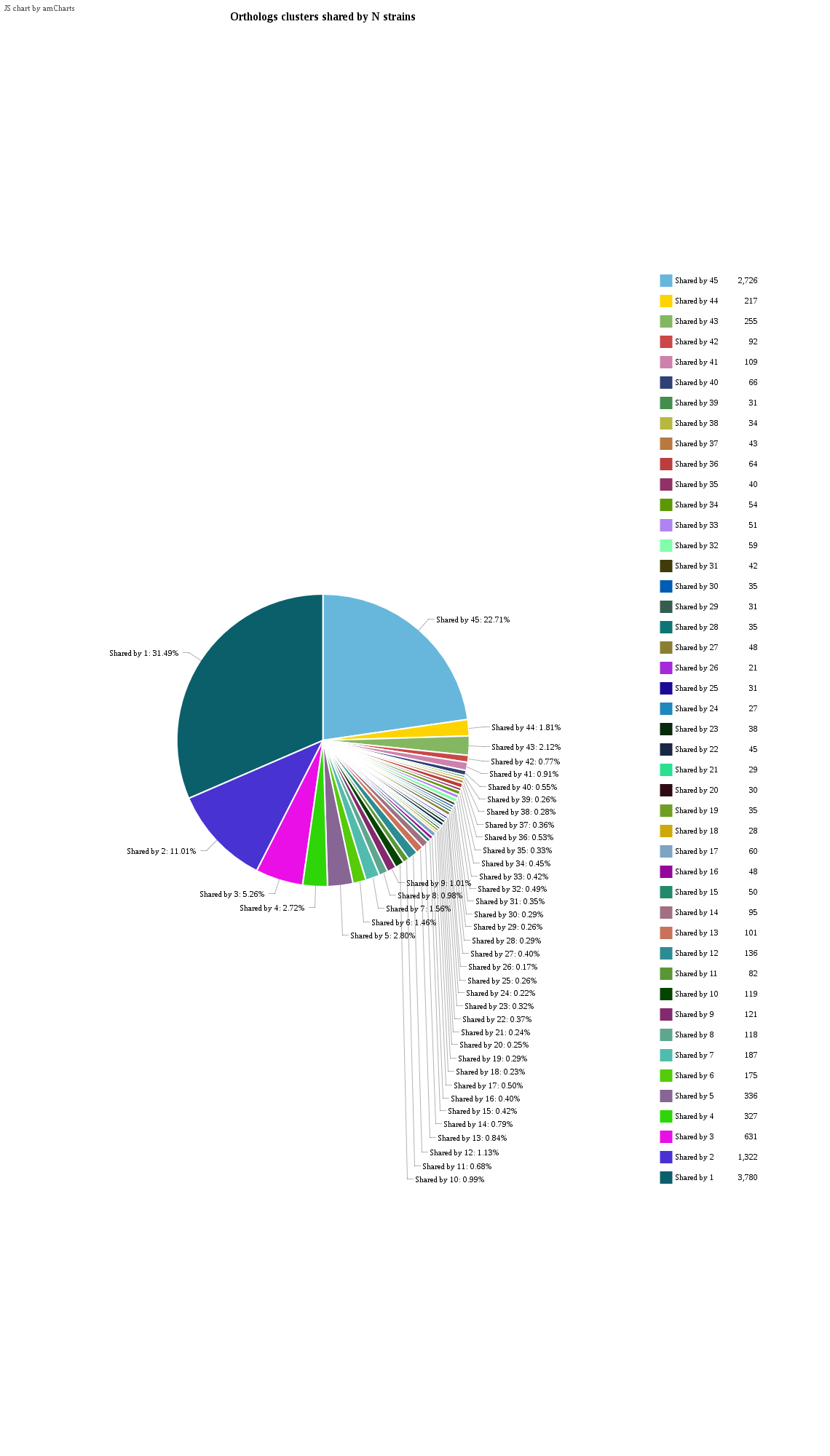

Supplement: S3 Fig — Pie charts showing the proportion of homologous genes shared among the n strains. (TIFF) [file pone.0178154.s003.tiff]

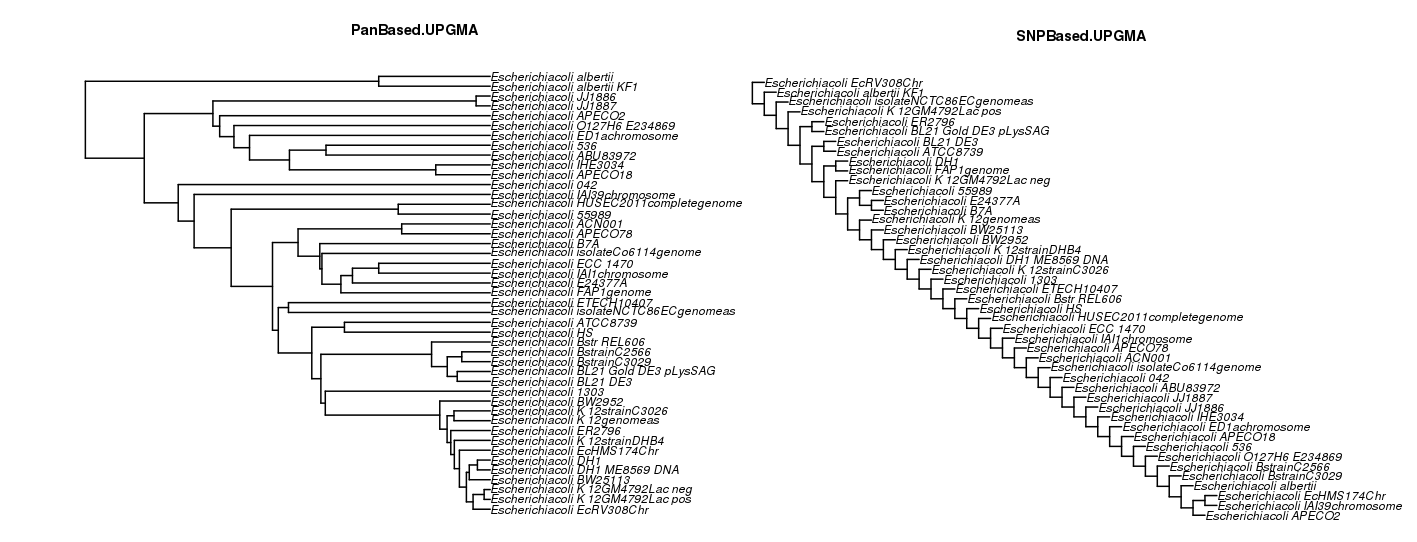

Supplement: S4 Fig — Phylogenetic trees showing species evolution analysis for the 45 strains based on the UPGMA algorithm. The left graph is based on the gene distance matrix for core gene clusters, and the right graph is based on indel variations in core-gene clusters. (TIFF) [file pone.0178154.s004.tiff]

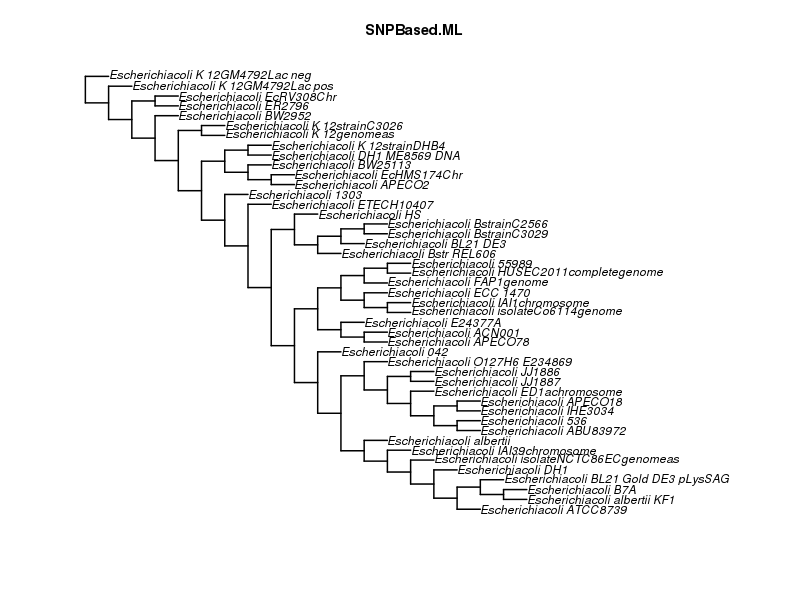

Supplement: S5 Fig — Phylogenetic tree showing species evolution analysis for the 45 strains based on the ML algorithm. The species evolution analysis is based on indel variations in core-gene clusters. (TIFF) [file pone.0178154.s005.tiff]
